# Olfaction in eating disorders and abnormal eating behavior: a systematic review

**DOI:** 10.3389/fpsyg.2015.01431

**Published:** 2015-09-30

**Authors:** Mohammed A. Islam, Ana B. Fagundo, Jon Arcelus, Zaida Agüera, Susana Jiménez-Murcia, José M. Fernández-Real, Francisco J. Tinahones, Rafael de la Torre, Cristina Botella, Gema Frühbeck, Felipe F. Casanueva, José M. Menchón, Fernando Fernandez-Aranda

**Affiliations:** ^1^Department of Psychiatry, University Hospital of Bellvitge-IDIBELLBarcelona, Spain; ^2^CIBER de Fisiopatologia de la Obesidad y Nutrición -ISCIIIBarcelona, Spain; ^3^Leicester Eating Disorder Service, Bennion Centre, Leicester Glenfield HospitalLeicester, UK; ^4^Division of Psychiatry and Applied Psychology, Faculty of Medicine and Health Sciences, University of NottinghamNottingham, UK; ^5^Department of Clinical Sciences, School of Medicine, University of BarcelonaBarcelona, Spain; ^6^Department of Diabetes, Endocrinology and Nutrition, Institut d'Investigació Biomèdica de Girona (IdlBGi) Hospital Dr Josep TruetaGirona, Spain; ^7^Department of Diabetes, Endocrinology and Nutrition, Hospital Clínico Universitario Virgen de VictoriaMálaga, Spain; ^8^Facultat de Ciencies de la Salut i de la Vida, Universitat Pompeu Fabra (CEXS-UPF)Barcelona, Spain; ^9^Integrative Pharmacology and Neurosciences Systems Research Group, Neuroscience Research Program, IMIM (Hospital del Mar Medical Research Institute)Barcelona, Spain; ^10^Department of Basic Psychology, Clinic and Psychobiology, University Jaume ICastelló, Spain; ^11^Department of Endocrinology and Nutrition, Clínica Universidad de Navarra, University of Navarra, IdiSNAPamplona, Spain; ^12^Endocrine Division, Complejo Hospitalario Universitario de SantiagoSantiago de Compostela, Spain; ^13^CIBER de Salud Mental (CIBERSAM)Madrid, Spain

**Keywords:** bulimia nervosa, anorexia nervosa, binge eating disorder, obesity, systematic review, olfaction, sniffin' sticks, abnormal eating

## Abstract

The study provides a systematic review that explores the current literature on olfactory capacity in abnormal eating behavior. The objective is to present a basis for discussion on whether research in olfaction in eating disorders may offer additional insight with regard to the complex etiopathology of eating disorders (ED) and abnormal eating behaviors. Electronic databases (Medline, PsycINFO, PubMed, Science Direct, and Web of Science) were searched using the components in relation to olfaction and combining them with the components related to abnormal eating behavior. Out of 1352 articles, titles were first excluded by title (*n* = 64) and then by abstract and fulltext resulting in a final selection of 14 articles (820 patients and 385 control participants) for this review. The highest number of existing literature on olfaction in ED were carried out with AN patients (78.6%) followed by BN patients (35.7%) and obese individuals (14.3%). Most studies were only conducted on females. The general findings support that olfaction is altered in AN and in obesity and indicates toward there being little to no difference in olfactory capacity between BN patients and the general population. Due to the limited number of studies and heterogeneity this review stresses on the importance of more research on olfaction and abnormal eating behavior.

## Introduction

Olfactory capacity has been widely studied in a range of mental health problems and its role in diagnosis and prognosis continues to be a significant research topic (Burón and Bulbena, 2013; Schecklmann et al., 2013). This capacity refers to the ability to smell (Hoover, 2010) and the existing literature has identified three commonly measured aspects of olfaction to be used as standardized measurements of olfactory functioning (Schecklmann et al., 2012): threshold (also known as sensitivity, Atanasova et al., 2008), discrimination and identification (also known as recognition, Dazzi et al., 2013). Olfactory threshold tests assess the minimum concentration of olfactory stimulus required in order for a person to smell (Atanasova et al., 2008). The biochemical pathway of this process begins at the nasal cavity and moves all the way to the olfactory bulb, which is located underneath the frontal lobe (Hoover, 2010). The discrimination test assesses the ability to differentiate one odor from another. The identification test requires the participants to be able to identify various odors and scents (Aschenbrenner et al., 2009). Both processes require the involvement of the limbic region (that includes the primary olfactory cortex, the amygdala–hippocampal complex, and the entorhinal cortex) where smell interacts with memory and emotion (Hoover, 2010). Both the hippocampus and the amygdala play a key role in the recognition of odors (Smitka et al., 2012).

The brain reward system is an active participant involved in eating behavior (Berridge, 2009) and reactions to olfactory stimuli (Frank et al., 2012). Pleasurable responses provoked primarily by olfactory and gustatory food stimulus (even though visual stimuli also have a key role) (Brunerová and Andel, 2014) have to be transferred to motivation or desire to eat in order for food reward to affect eating behavior (Berridge, 2009; Frank et al., 2012). Distortions of this reward system may be a contributing factor in ED (Berridge, 2009). Animal and some human studies imply that while food restriction can sensitize the brain reward pathways, excessive food intake can desensitize it (Frank et al., 2012). Studies on overweight rats have shown alterations in olfactory capacity to be stronger (Badonnel et al., 2014) and weaker (Thiebaud et al., 2014). The orbitofrontal cortex, a key brain area of the reward circuit (of the brain reward system), has been found to be more responsive to food stimuli in AN (anorexia nervosa), while it is the opposite case in obese women where activation of the orbitofrontal cortex is highly reduced (Frank et al., 2012). Olfactory dysfunction was found to strongly affect both ingestive and dietary behavior such as changes in weight, loss of pleasure in taste and decrease in food intake (Aschenbrenner et al., 2008) and appetite (Hummel and Nordin, 2005; Stevenson, 2010). Thus, given its significance in eating behavior, it can be hypothesized that olfaction may influence eating behavior.

Previous studies have shown smell dysfunction in a number of major psychiatric disorders, including schizophrenia, obsessive-compulsive disorder, depression, and dementia (Atanasova et al., 2008; Rotge et al., 2009; Segalàs et al., 2011; Calkins et al., 2013; Schecklmann et al., 2013). However, the few studies that investigated the association between olfactory capacity and eating disorders (ED) have reached conflicting conclusions due to methodological limitations (namely lack of sample power and discrepancies in the assessment procedures used) (Richardson et al., 2004; Lombion-Pouthier et al., 2006; Schreder et al., 2008; Rapps et al., 2010; Weiland et al., 2011; Stein et al., 2012; Dazzi et al., 2013).

The aim of the present study is to provide a systematic review that explores the current literature on olfactory capacity in abnormal eating behavior. It is thus intended to present a basis for discussion on whether research on olfaction in ED may provide additional insights concerning the complex etiopathology of ED and abnormal eating behaviors.

## Methods

### Search strategy

**Figure 1:**
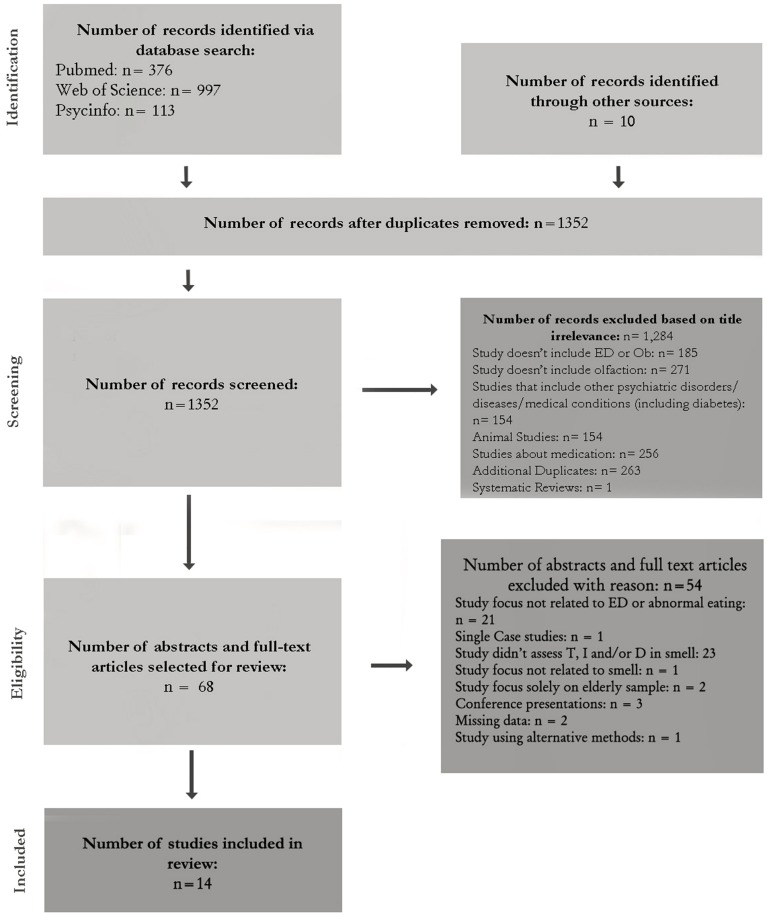
**Flow chart of literature search**.

Electronic databases (Medline, PsycINFO, PubMed, Science Direct, and Web of Science) were searched using the following five components in relation to olfaction in all possible permutations: olfaction (olfactory), smell, odor, anosmia, sniffin' sticks. First using the OR operator, and then using the AND operator, these search terms were combined with the following components related to EDs [body-mass, eating disorder, abnormal eating, anorexia nervosa, bulimia nervosa, obesity, binge-eating disorder, eating disorder not otherwise specified (EDNOS)]. Databases were searched from 1966 until November 2014. The search strategy is shown in Figure 1. The data were extracted by independent authors (MAI and ABF). Discrepancies or disagreements were discussed with an independent researcher (IS). A full text version was obtained for all studies identified for inclusion. All studies were reviewed with regard to their quality and eligibility for the review.

Figure 1 outlines the search steps and Table 1 illustrates the selection criteria. The importance of the articles yielded by the combined search keys in Pubmed (*n* = 376), Web of Science (*n* = 997), and Psycinfo (*n* = 113) were further ascertained by screening titles, abstracts and, in some cases, the full-text articles themselves. Other sources of search included the archives of the University Hospital of Bellvitge. Where fulltext articles were not available directly from the online databases or at the university, the authors were contacted for a copy.

**Table 1:** **Selection criteria for the studies included in this review**.

**Category**	**Criteria**
Study population	All ethnicities All ages (except studies **only** assessing elderly humans) General population, college student populations and clinical samples (with ED or Abnormal eating behavior and with no neurologic and/or psychiatric disorders or medical diseases) Males and females
Study geography	All nations
Language	English, French, Spanish, German, and Italian
Period	1966-June 2014
Type of studies	Human studies (clinical and community); Qualitative studies; case control studies; designs of an experimental nature; no single case designs; studies specifically measuring olfactory threshold, discrimination, and/or identification; no book chapters, dissertations, or studies about effects of medication

This systematic review employed the NICE rating system to check the methodological quality of studies (NICE, 2007; NICE, see Table 2). The NICE rating system rates the studies as very good quality (where all or most of the criteria have been fulfilled) (++), adequate quality (where some of the criteria have been fulfilled) (+), and poor quality (where few or no criteria have been fulfilled (−). The score is presented on the far right side of Table 3.

**Table 2:** **NICE rating system for methodological quality of studies using methodological checklists (NICE, 2007)**.

**++**	All or most of the criteria have been fulfilled. Where they have not been fulfilled the conclusions of the study or review are thought **very unlikely** to alter
+	Some of the criteria have been fulfilled. Those criteria that have not been fulfilled or not adequately described are thought **unlikely** to alter the conclusions
−	Few or no criteria fulfilled. The conclusions of the study are thought **likely or very likely** to alter

**Table 3:** **Studies of olfaction in eating disorders and abnormal eating (sorted by disorder)**.

**Authors**	**Participants**			**Design of study**	**Methods**		**Results**				**Comments**	**Score**
	**Sample**	**Mean Age (years) (sd)**	**Not controlled for/no information with regard to**		**Olfactory Tests**	**ED Criteria/Symptoms**	**OT Mean (sd)**	**OD Mean (sd)**	**OI Mean (sd)**	**TDI Mean (sd)**	**Interpretation of Results**	
**ANOREXIA NERVOSA**												
Kopala et al., 1995	AN = 27 HC = 50, females	Range 14–29 AN: 20.1 (3.6) HC: 27.4 (11.1)	Hyposmia/anosmia in HC	Case-control	UPSIT	DSM-III-R, DSM-IV			AN = 38.5 (*sd* = 1.4) HC = 38.2 (*sd* = 1.3)		No significant differences were found between HC and AN group in olfactory identification	+
Lombion-Pouthier et al., 2006	AN = 17 F HC = 36F + 22M	AN: 22.7, *sd* = 6.8 HC: 38.4; sd = 13.9.	Medication, smoking/Hyposmia or anosmia in HC	Case-control	Test Olfactif	DSM-IV	AN = 2.7 (1.4) HC = 3.6 (1.3)			n/a	AN scored higher in threshold	+
Schreder et al., 2008	AN = 12 HC = 22 females	AN: Range 17–27, *m* = 20.2 (*sd* = 3.2) HC: Range 20–30, *m* = 24.1 (*sd* = 2.6)	(Mild) depression	Case-control	Sniffin' Sticks, SAM,	DSM-IV, SIAB-S,	AN: 10.17 (*sd* = 2.0) HC: 10.2 (*sd* = 1.9)	AN: 12.5 (1.5) HC: 13.7 (1.4)	AN:13.3 (*sd* = 1.3) HC: 14.4 (*sd* = 0.7)	AN: 35.38 (*sd* = 3.88) HC: 38.47 (*sd* = 2.50)	Patients scored significantly lower than HC in odor discrimination, identification tests and TDI	+
Rapps et al., 2010	9 AN (BP), 10 AN (RT). 21 HC females	AN: 22, *sd* = 3. HC: 22, *sd* = 2)	levels of depression + medication (anti-depressants)	Case-control	Sniffin' sticks	DSM-IV, EDI-2	AN = 8.6 (*sd* = 2.1) HC = 9 (*sd* = 1.7)	AN = 12.2 (*sd* = 1.6) *HC* = 13 (*sd* = 1.7)	AN = 12.9 (*sd* = 1.5) HC = 13.9 (*sd* = 1.2)	AN = 33.8 (*sd* = 3.2) HC = 34.9 (*sd* = 4.2)	OT AN = HC OD AN = HC OI AN < HC TDI AN = HC	+
Goldzak-Kunik et al., 2012	14 female + 1 male AN patients HC (14F, M1)	AN: mean = 15.8, *sd* = 0.3 HC: mean = 15, *sd* = 0.4	Hyposmia/anosmia in HC/medication	Case-control	Sniffin' Sticks (sensale)	EDI	NA	n/a	NA	n/a	OT AN = HC OI AN > HC	+
Schecklmann et al., 2012	26 AN patients + 23 HC, females	Range 9–18 AN: 15.5, (*sd* = 1.8) HC: 14.7 (*sd* = 2.5)	Hyposmia/anosmia in HC	Case-control	Sniffin' Sticks	DSM-IV, ICD-10, EDI-2	AN = 9.9 (*sd* = ±2.3) HC = 9.6 *sd* = ±2.3)	AN = 12.4 (*sd* = ±2.0) HC = 11.8 ± (*sd* = 1.9)	AN = 12.9 (*sd* = ±1.9) HC = 12.0 (*sd* = ±1.8)		Only identification of smell was found to be significantly superior in AN group	+
**ANOREXIA NERVOSA, BULIMIA NERVOSA, EDNOS**												
Fedoroff et al., 1995	25 AN-R 15 AN-BP 15 BN 16HC female	ED: Range 12–46 (mean = 24.1, *sd* = 1.1) HC: Range 12–46 (mean = 25.8, *sd* = 1.2	Hyposmia/anosmia in HC, smoking	Case-control	UPSIT, PEA test	DSM-III-R, EAT, Eating Inventory	ANR > 70%BW –7.8 (0.6) ANR < 70%BW –7.3 (0.6) AN-BP: –8.8 (0.6) BN: –9.6(0.6) HC: –8.2 (0.6)	n/a	AN(B) 37.6 (0.4) BN 38.3 (0.5) ANR > 70% = 38.4 (*sd* = 0.6) ANR < 70% = 35.3 (*sd* = 2.0) HC = 38.4 (*sd* = 0.3)	n/a	Low-weight AN showed impairments in their ability to identify and detect odors	+
Aschenbrenner et al., 2009	16 AN 24 BN 23 HC females	Adults AN Range 19–32 (mean = 24.5, *sd* = 4), BN Range 19–35 (mean = 24.3, *sd* = 4.6), HC Range 18–34 (mean = 24.5, *sd* = 4.8)	Hyposmia/(anosmia in HC, medication, depression	Case-control,	Sniffin' Sticks	DSM-IV, EAT, DIA-X/M-CIDI	AN = 9.2 (*sd* = 1.6) BN = 10.0 (*sd* = 2.0) HC = 10.1 (sd = 2.3)	(AN) – (BN) = AN = 13.2 (sd = 1.9) BN = 14.3 (sd = 1.0) HC = 14.7 (sd = 1.0)	= AN = 14.1 (sd = 1.1) BN = 14.2 (sd = 0.9) HC = 14.3 (sd = 1.1)	AN = 36.6 (sd = 2.7)- BN = 38.6 (sd = 2.7) HC = 39.3 (sd = 2.3)	AN have significantly lowered olfactory function than BN and HC. Total average (TDI) is lower in patients with AN	++
Stein et al., 2012	40 AN (RT), 23 AN (BP), 20 BN, 13 EDNOS, 20 HC females	AN-RT = 15.6±1.7, AN-BP = 16.3±1.3, BN and EDNOS = 16.6±1.3	Medication (SSRI), patients with depression and anxiety disorders (except OCD), anosmia/hyposmia in HC	Case-control	Three-bottle olfactory test (and: DSM-IV, EDFHI, BDI, Y-BOCS, YBC-ED and alternation learning task)		AN-R = 6.4, *sd* = 1.4 AN-BP = 6.7, *sd* = 1.1 BN/EDNOS = 6.7, *sd* = 0.9 HC = 7.5 (*sd* = 1.1)	+ AN-R = 50.6, *sd* = 6.4 AN-BP = 51.5, *sd* = 5.2 BN/EDNOS = 48.2, *sd* = 9.0 HC = 45.5, *sd* = 9.1	N/A	n/a	The eating disorder group scored significantly lower for threshold and significantly higher for discrimination	+
Dazzi et al., 2013	18 AN + 19 BN female patients + 19 HC (5M+14F)	Range 16-47 (mean = 26.5, sd = 6.2	Education, other psychopathy, medication, 5% of HC with hyposmia	Case-control	Sniffin' Sticks	EDI-2, DSM-IV	AN = 6.3 (sd = 4.0) BN = 5.5 (sd = 3.9) HC = 9.1 (sd = 1.3)	- AN = 11.6 (*sd*=1.8) BN = 12.0 (*sd* = 1.9) HC = 13.5 (*sd* = 0.8)	= AN = 11.6 (*sd* = 1.9) BN = 10.7 (*sd* = 2.2) HC = 11.8 (*sd* = 1.4)	– AN = 29.5 (*sd* = 5.6) BN = 28.1 (*sd* = 6.4) HC = 34.5 (*sd* = 2.3)	No significant differences in identification. BN have lower threshold than HC. AN and BN lower discrimination than HC. Total average (TDI) is lower in patients with ED indicating hyposmia	+
**BULIMIA NERVOSA**												
Weiland et al., 2011	12 BN, 12 HC females	Adults BN = 24, (*sd* = 3.6) HC = 32 (*sd* = 7.9)	Smoking, age (HC significantly older than BN). Dopaminergic meds	Case-control	Sniffin' Sticks with n-butanol sensitivity		HC = 8.2 (*sd* = 1.6) BN = 8.3 (*sd* = 0.9),	N/A	N/A		The scores for threshold did not significantly differ between BN group and controls	+
**OVERWEIGHT/OBESITY**												
Simchen et al., 2006	311 patients (58% females, 42% males)	Range 20–88	Excludes participants younger than 20, age group only distributed as 65+ and 65-	Cross-sectional (without control)	ETOC	Standardized questionnaire, BMI	–(in < 65 group, BMI > 28 = lower score (±15.3 as opposed to ±15.5 in BMI < 28 group). +(in > 65 group, BMI > 28 = higher score (±14.6 as opposed to ±13.8 in BMI < 28 group)		–(in < 65 group, BMI > 28 = lower score (± 12.2 as opposed to ±13 in BMI < 28 group) + (in > 65 group, BMI > 28 = higher score (±10.9) as opposed to ±10.2 in BMI < 28 group)		Scores for odor detection and odor identification were lower in participants with a BMI > 28 kg/m2 than in participants with a BMI < 28 kg/m2 when the age was < 65 years, whereas in participants > 65 years scores were higher in participants with a BMI > 28 kg/m2 than in participants with a BMI < 28 kg/m2 (BMI^*^age group: P¼0.015, 0.053, and 0.015, respectively). There was no control group	+
**OBESITY**												
Obrebowski et al., 2000	15 boys and 15 girls undergoing rehabilitation	12 (Range 10–16)	No control group	Cross-sectional (without control)	Elsberg–Levy's olfactometry, Electrogustometric tests		– Lower score (1.6–3 cm^3^) in 43% of sample for natural coffee and 63% for anise oil compared to mean = 1.5 cm^3^			N/A	Scores show very low levels of olfactory threshold, below the mean	+
**OBESITY AND ANOREXIA NERVOSA**												
Fernández-Aranda et al., 2015	64 AN 80 young HC 36 old HC 59 obese females	AN = 24.0 (*sd* = 5.3) Young HC = 22.6 (*sd* = 2.9) Old HC = 37.3 (5.9) Ob = 37.5 (8.7)		Case control	Sniffin' Sticks	DSM-IV, EDI-2, SCL 90-Revised and DEBQ	AN = 8.7 (*sd* = 0.3) yHC = 6.4 (*sd* = 0.3) oHC = 7.1 (*sd* = 0.4) Ob = 5.9 (*sd* = 0.3 AN > HC	AN = 13.0 (*sd* = 0.3) yHC = 12.5 (*sd* = 0.2) oHC = 13.2 (*sd* = 0.3) Ob = 12.5 (*sd* = 0.3)	AN = 13.0 (*sd* = 0.3) yHC = 13.1 (*sd* = 0.2) oHC = 12.8 (*sd* = 0.3) Ob = 11.8 (*sd* = 0.3	AN = 34.8 (*sd* = 0.6) yHC = 32.0 (*sd* = 0.5) oHC = 33.1 (*sd* = 0.8 Ob = 30.3 (*sd* = 0.6) AN > HC	Superior odor threshold in the AN group compared to other groups, and also better odor identification than obese group. Obese individuals showed the poorer olfactory capacity and the greater prevalence of hyposmia	+

*M, males; F, females; AN, anorexia nervosa; BN, bulimia nervosa; ED, eating disorders; HC, human control; RT, restricting type; BDI, Beck Depression Inventory; BP, binge purge type; CBCL, Child Behavior Checklist; DIKJ, a quantitative self-rating scale for depression; EAT, Eating Attitude Test; EDNOS, eating disorder not otherwise specified; DIA-X/M-CIDI, Munich-Composite International Diagnostic Interview; EDFHI, Eating Disorders Family History Interview; ETOC, European Test of Olfactory Capabilities; Ob, Obese; PEA, phenyl ethyl alcohol; SAM, Self-Assessment Manikin; SIAB-S, Structured Inventory for Anorexic and Bulimic Eating Disorders; UPSIT, University of Pennsylvania Smell Identification Test; Y-BOCS, Yale-Brown Obsessive Compulsive Scale; YBC-ED, Yale-Brown-Cornell Eating Disorders Scale; MMPI-2, Minnesota Multiphasic Personality Inventory-2; EDI-2, Eating Disorder Inventory-2*.

There are other factors that ought to be taken into consideration when assessing the results. Age (Hudson et al., 2012; Sorokowska et al., 2015) and gender (Doty and Cameron, 2009; Malaspina et al., 2012) have been found to be associated with changes in olfaction. It has been found to be higher in adults (up to a certain age) and women (Mullol et al., 2012). Smoking has also found to strongly affect olfactory capacity (Hayes and Jinks, 2012; Gudziol et al., 2013; Moccia et al., 2014). In chronic smokers, it might even lead to permanent hyposmia (Gudziol et al., 2013). Alterations in olfactory capacity have also been found observed in individuals who: took medication, namely antidepressants (Lombion et al., 2008), have diabetes (Gouveri et al., 2014; Mehdizadeh et al., 2015), and have depression (Negoias et al., 2010). Type of patient (whether inpatient or outpatient) might also affect the results as inpatients tend to have a more severe illness.

### Summary of the studies

The total number of articles obtained from the combined search keys was 1352. Studies meeting the inclusion criteria were first examined by titles. This yielded 68 studies. Titles that did not imply any connection between EDs or abnormal eating and smell or that examined comorbidity with other psychiatric and medical illnesses or effects of medication were rejected. The next step involved reading abstracts or fulltexts of the 68 articles. Those that did not meet the criteria were rejected. Studies that only included an elderly sample were excluded because old age (cutoff was at 60 years) was found to influence olfaction (see Figure 1). After excluding 54 studies, a final total of 14 studies were selected.

The Results Section will describe first the characteristics of the studies, followed by a description of the methods used to assess olfaction in the literature. As the principle aim of the paper is to review the findings of olfactory capacity in various EDs, the last part of the Results Section will present the findings of the review grouped according to the abnormal eating behavior and ED diagnostic categories.

## Results

### Characteristics of the studies

Table 2 outlines the 14 articles selected for this review. Eight (57.1%) of them were published in the last five years. Six (42.9%) from 2010 to 2013, four (28.6%) between 2006 and 2009, two (14.3%) of the total studies are from the 1990s and two (14.3%) from 2000 to 2004 (see Table 2). The total sample added up to study participants (including patients) and 385 control participants. Out of the 14 articles, 11 (78.6%) included a female sample only. All studies were published in English.

Eight (57%) studies assessed a single disorder and 6 (43%) studied more than one disorder (mainly anorexia nervosa, bulimia nervosa and/or obesity). Eleven (78.6%) of the studies included participants with AN, five (35.7%) with BN, two (14.3%) with obesity, one (7.1%) who were overweight (BMI between 25 and 30) and one (7%) with EDNOS. The search did not identify olfaction studies conducted on individuals with BED (binge-eating disorder).

The criteria of the NICE checklist (NICE, 2007) are illustrated in Table 2. One study (Aschenbrenner et al., 2009) scored (++) because it fulfilled most of the criteria and its potential replicability of results was believed to be high. Despite this, it still contained some flaws e.g., low sample power (*n* = 63), not controlling for hyposmia and depression. The rest of the 13 studies (Fedoroff et al., 1995; Kopala et al., 1995; Obrebowski et al., 2000; Lombion-Pouthier et al., 2006; Simchen et al., 2006; Schreder et al., 2008; Rapps et al., 2010; Weiland et al., 2011; Goldzak-Kunik et al., 2012; Schecklmann et al., 2012; Stein et al., 2012; Dazzi et al., 2013; Fernández-Aranda et al., 2015) scored (+) because it is believed that they fulfilled at least some of the criteria where the conclusion is unlikely to change upon repetition. The findings of these studies are further explored in the following sections.

### Measuring olfaction

The Sniffin' Sticks test (Hummel et al., 1997) was used in eight (57%) studies published between 2008 and 2014 (Schreder et al., 2008; Aschenbrenner et al., 2009; Rapps et al., 2010; Weiland et al., 2011; Goldzak-Kunik et al., 2012; Schecklmann et al., 2012; Dazzi et al., 2013; Fernández-Aranda et al., 2015) (see Table 3). This test is divided into three categories: the olfactory threshold test, the discrimination test and the identification test. The olfactory threshold test helps determine the minimum level of odor one can detect. This has been linked to food reward (McNeil et al., 2013). Discrimination and identification too have been associated with food reward as they further play parts in food selection and preference (Simchen et al., 2006; McNeil et al., 2013).

The olfactory threshold test consists of a series of 16 rows of three odor pens placed in ascending order (from weakest concentration to strongest concentration, each row consisting of a different concentration). In each trial, the three odor pens of the same row are presented to the blindfolded participant in random order where only one odor pen contains the concentrated odorant and the other two remain odorless. One by one, the odor pens are placed in front of the participant's nostrils (within a 2 cm distance) and asked to identify the odor pen containing the odor. Administrator starts noting the score after the participant makes the first successful detection, the higher the concentration, the lower the score. Therefore, higher score indicates more sensitivity. Each time the participant correctly detects an odor the administrator records it. After six successful trials, the average score of the last four are noted.

The discrimination test follows a similar procedure except that here two of the three pens have the same odor and one has a different one. The participant is asked to identify the odor pen that smells differently. The total score is calculated based on the total number of pens the participant was able to differentiate correctly. The identification test consists of 16 odor pens, each containing a different odorant. Each odor pen is placed in front of the participant's nostrils. The participant is asked to identify the odor and is then presented with a card that lists 4 objects. Participant chooses the object that smells like the odor pen. The sum of correct identifications leads to the total score. The overall olfactory capacity (TDI, ranging from 1 to 48) is obtained by calculating the sum of the scores of the three tests and then matched with a checklist.

The UPSIT (University of Pennsylvania Smell Identification Test) (Doty et al., 1984) was found to be highly reliable (Doty et al., 1989; Fedoroff et al., 1995) and responsive to changes in olfactory functioning (Fedoroff et al., 1995). However, this standardized 40-item multiple choice test only detects one's capacity to identify odors as opposed to discrimination and detection.

The ETOC (European Test of Olfactory Capabilities) (Thomas-Danguin et al., 2003) applied 16 series of four vials each. Only one of the four vials contained an odor, all of which were different. The participant's task was to detect which of the four vials had an odor and then to identify the odor. The test was found to be highly reliable but it only measured threshold and identification (Thomas-Danguin et al., 2003; Simchen et al., 2006).

A PEA (Phenyl Ethyl Alcohol) test was used to measure olfactory threshold in one (Fedoroff et al., 1995) study (7%) which also used the UPSIT. This was done using a “single case forced choice paradigm incorporating PEA in a half-log dilution series extending from −13 to −1 log concentrations” (Fedoroff et al., 1995, p. 73). The participant's task was to state which pair of stimulus provoked the stronger response. However, its use is not explained in detail and its reliability is not known. One study (Welge-Lüssen et al., 2003) found it to be less reliable than another test [namely a CO(2) test].

The Elsberg Levy's olfactometry (Pruszewicz, 1965) was used to measure olfaction by employing (Obrebowski et al., 2000) coffee, anise oil, lemon, and peppermint as odorants. This is an old test that only measures olfactory threshold and identification and it was modified by Pruszewicz in 1965.

The Test Olfactif was used in one study (7%) in France (Lombion-Pouthier et al., 2006). It assesses olfactory threshold, detection, and identification for 16 different odors. For olfactory threshold the test uses a forced choice procedure for 5 consecutive concentrations of l-carvone and tetrahydrothiophene. However, considering that very little is known about this test, there's no mention of its reliability nor does the search in databases yield many results.

A three-bottle olfactory test was used in one study (7%) (Stein et al., 2012). Olfactory threshold was measured by a series of three-way forced choice trials. Respondents were presented with three bottles. Two of them had the same odorant whereas the third had a different one. They were asked to identify the bottle containing the different odorant. The “lower of two consecutive concentrations for which four consecutive correct detections were obtained” (Stein et al., 2012, p. 618) determined the threshold. In the discrimination test five different odorants were used. Of these odorants there were 60 trios. In each trial participants were asked to choose between three odorants (one was a given odorant and two contained a second one). Aside from the fact that this test only measures threshold and discrimination, it is very much a homemade test that has not been assessed for reliability.

While there are tests that measure the three aspects of olfaction (threshold, discrimination and identification) separately, the “Sniffin' Sticks” (Hummel et al., 1997) test is the only standardized and validated test that measures all three and it has been used to assess olfactory capacity in a number of studies on patients with neurological disorders and healthy controls (Segalàs et al., 2011). Of all the tests used in these studies, this is the most complete one.

## Olfaction in abnormal eating behaviors

### Anorexia nervosa

The majority of research on olfaction and EDs was conducted in AN (*n* = 11). Three (21.4%) of those articles had investigated both AN and BN (Fedoroff et al., 1995; Aschenbrenner et al., 2009; Dazzi et al., 2013), one (7.1%) had looked at AN, BN, and EDNOS (Stein et al., 2012) and one (7.1%) looked at AN and obesity (Fernández-Aranda et al., 2015). The AN studies amount to a total sample of 317 AN patients and 383 healthy controls (377 females, 6 males). All studies followed a case control design. In Schreder et al. (2008), Aschenbrenner et al. (2009), Rapps et al. (2010), Goldzak-Kunik et al. (2012), Schecklmann et al. (2012), Dazzi et al. (2013), and Fernández-Aranda et al. (2015) the Sniffin' Sticks test (Hummel et al., 1997) was used. In Fedoroff et al. (1995) and Kopala et al. (1995) the UPSIT (Doty et al., 1984) was applied. In Fedoroff et al. (1995) a PEA test was also used. In Lombion-Pouthier et al. (2006) the Test Olfactif was used and in Stein et al. (2012) a three-bottle olfactory test was administered. After 2009, all olfactory studies on AN, with the exception of one (Stein et al., 2012), used the Sniffin' Sticks test.

#### Olfactory threshold

In 3 of the 11 studies (27.3%) clinical patients with AN had a lower olfactory threshold than controls (Fedoroff et al., 1995; Aschenbrenner et al., 2009; Stein et al., 2012). One study (Lombion-Pouthier et al., 2006) that used the Test Olfactif (a French olfactory test) shows the opposite result where AN scored higher than controls. Another study (Fernández-Aranda et al., 2015) that used the Sniffin' Sticks test also showed similar results where patients scored significantly higher than young controls [age range: 19–29 (average age 22.6, *sd* = 2.9), old controls (age range: 30–50 (average age 37.3, *sd* = 5.9)] and an obese group. What is further noted here is that the threshold score of the controls is lower (6.42, *sd* = 0.30) than that of the controls in other studies where the score ranged from 7.5 to 13.75 (see Table 3). However, this is the only study (see Table 3) that controlled for the presence of hyposmia or anosmia in the control group, screened medication use, smoking and comorbidity and it has the highest sample power (64 patients, twice the size of most). Five studies (Schreder et al., 2008; Rapps et al., 2010; Goldzak-Kunik et al., 2012; Schecklmann et al., 2012; Dazzi et al., 2013) showed no significant difference in olfactory threshold between the AN group and controls.

There could be a number of factors that affect olfaction and play a confounding role in influencing the results. The sample power in Lombion-Pouthier et al. (2006) and Aschenbrenner et al. (2009) was very low (*n* = 16 and *n* = 17, respectively). The latter study did not match gender as the control group included males (37.9%) whereas all 17 AN patients were females. Age of sample also varied. In Fedoroff et al. (1995), the sample age varied from 12 to 46 making it heterogeneous. Whereas in Aschenbrenner et al. (2009) it varied from 18 to 35. The age of AN patients in the study by Fernández-Aranda et al. (2015) ranged from 17 to 34 (average age 24, *sd* = 5.3). This makes the samples heterogeneous. Moreover, this article doesn't explain on what basis the age limit was set for the two control groups. In Aschenbrenner et al. (2009) and Stein et al. (2012), patients also took medication and some of them were found to suffer from depression. Smokers were included in the Fedoroff et al. (1995) and Lombion-Pouthier et al. (2006) samples. In the latter study it was also mentioned that the duration of the starvation period may have decreased cell renewal of the olfactory epithelium is and this could be a possible factor that has influenced the olfactory outcome. With the exception of Fernández-Aranda et al. (2015), all four of the above studies included in-patients in their sample which could lead to interpretational bias as it excludes out-patients and does not consider the effect the specific hospital environment might have. Moreover these patients tend to have a more severe disorder.

#### Olfactory discrimination

Four studies (Lombion-Pouthier et al., 2006; Rapps et al., 2010; Schecklmann et al., 2012; Fernández-Aranda et al., 2015) found no significant differences in olfactory discrimination between AN patients and controls. One study (Stein et al., 2012), that used a three-bottle olfactory test, showed olfactory discrimination to be higher in restrictive AN as well as in binge-purge type than in controls. However, three studies (Schreder et al., 2008; Aschenbrenner et al., 2009; Dazzi et al., 2013), all of which used the Sniffin' Sticks test, scored significantly lower in olfactory discrimination than healthy controls.

It ought to be mentioned that in Aschenbrenner et al. (2009) and Dazzi et al. (2013) there was no control for medication. In Schreder et al. (2008) and Aschenbrenner et al. (2009) participants suffering from depression were included in the sample. In Schreder et al. (2008), the patient group included inpatients and patients from self-help groups. The mean BMI of controls was 20.99 (*sd* = 1.71) and of the AN group it was 16.88 (*sd* = 1.26). However, Stein et al. (2012), used respondents who suffered from depression and anxiety disorders (including OCD) and who took medication. These factors could have a confounding effect. The advantage that this last study has over the other three is its high sample power (*n* = 116). Yet, as mentioned earlier, the validity of the test in Stein et al. (2012) also remains uncertain.

#### Olfactory identification

No significant differences were found in olfactory identification in Kopala et al. (1995), Lombion-Pouthier et al. (2006), Dazzi et al. (2013), and Fernández-Aranda et al. (2015). AN patients scored significantly better in olfactory identification in Goldzak-Kunik et al. (2012) and Schecklmann et al. (2012) than in controls. On the contrary, using the UPSIT, Fedoroff et al. (1995) showed low weight AN patients to score worse than controls. Scores were also significantly lower than controls in Schreder et al. (2008), Aschenbrenner et al. (2009), and Rapps et al. (2010).

The sample size in Schreder et al. (2008), Aschenbrenner et al. (2009), and Rapps et al. (2010), was very small. Patients in Goldzak-Kunik et al. (2012) and Schecklmann et al. (2012) in both groups were very young. In Schecklmann et al. (2012), a study which was conducted on adolescents, participants were as young as nine. Thus, developmental changes may have affected the results. In Fedoroff et al. (1995) the sample was too heterogeneous, mixing adults, and adolescents. This study included smokers in its sample. In Goldzak-Kunik et al. (2012), there is no mention of participants' smoking habits (whether this was controlled for or not). Some participants in both Goldzak-Kunik et al. (2012) and Schecklmann et al. (2012) took medication.

#### Overall olfactory capacity

Of the five studies that reported TDI (the total score of olfactory threshold, discrimination and identification calculated in the Sniffin' Sticks test), scores were significantly lower than controls in Schreder et al. (2008), Aschenbrenner et al. (2009), and Dazzi et al. (2013), and significantly higher in AN in Fernández-Aranda et al. (2015).

Compared to other EDs, anorexia nervosa shares the most number of studies with olfactory capacity. However, due to different olfactory procedures used the studies are too heterogeneous for a meta-analysis. Stafford et al. (2013) found that increases in pathological eating attitudes in a student sample predicted poorer responses to olfactory stimulus in a non-clinical sample. Even though a solid conclusion cannot be drawn due to the limited number of studies showing opposing results, the overall findings seem to indicate toward a weaker olfaction in AN. Yet, what is interesting here is how Fernández-Aranda et al. (2015) study observes a stronger olfactory capacity in AN. Despite contradicting most of the findings of other studies, Fernández-Aranda et al. (2015) provides the strongest results based on statistical power and for controlling of possible confounding variables. However, this one study alone is not sufficient to draw a precise conclusion in a systematic review.

### Bulimia nervosa

A total of 5 studies analyzed olfactory function in patients with BN (bulimia nervosa) (Fedoroff et al., 1995; Aschenbrenner et al., 2009; Weiland et al., 2011; Stein et al., 2012; Dazzi et al., 2013). Three of the studies used the Sniffin' sticks to test olfactory function (Aschenbrenner et al., 2009; Weiland et al., 2011; Dazzi et al., 2013), one (Fedoroff et al., 1995) used the UPSIT (Doty et al., 1984). The total sample of both the patient group and healthy controls adds up to 90 individuals each. With the exception of 5 males in the control group of the study conducted by Dazzi et al. (2013), the participants were females.

#### Olfactory threshold

All five studies tested the olfactory threshold of participants. Three (Fedoroff et al., 1995; Aschenbrenner et al., 2009; Weiland et al., 2011) showed no significant differences with respect to controls. Two studies (Stein et al., 2012; Dazzi et al., 2013) showed threshold to be significantly lower than controls. It is noted that in one of the two studies, BN patients were combined with EDNOS patients (Stein et al., 2012). In four studies (Fedoroff et al., 1995; Aschenbrenner et al., 2009; Stein et al., 2012; Dazzi et al., 2013) a comparison was also made with an AN group but olfactory threshold showed no significant differences.

#### Olfactory discrimination and identification

Of the three BN studies that tested olfactory discrimination, one (Dazzi et al., 2013) showed this capacity to be significantly lower in BN compared to the control group while another (Stein et al., 2012), where BN patients were combined with EDNOS, the capacity was significantly higher in the patient group. The third study (Aschenbrenner et al., 2009) showed no statistically significant differences in relation to the control group. However, BN patients did score significantly higher than AN patients. The three studies that tested olfactory identification (Fedoroff et al., 1995; Aschenbrenner et al., 2009; Dazzi et al., 2013) showed no significant differences compared to healthy controls).

#### Overall olfactory capacity

The difference in the TDI score in Aschenbrenner et al.'s (2009) study was not significant while in Dazzi et al. (2013) the score was significantly lower in bulimics (28.18, *sd* = 6.43, which falls under hyposmia) compared to controls.

In the latter study the control group had 19 participants of which more than 25% were males while the patient group had 19 females in both the AN group and BN group. This heterogeneity could have affected the results as studies have shown gender differences in olfaction (Doty and Cameron, 2009; Malaspina et al., 2012). Moreover this study did not control for comorbidity (with other psychopathology) and medication which could be confounding factors. 57.8% of the BN group tested positive for hyposmia compared to 5% of controls in Dazzi et al. (2013).

Flaws of the study conducted by Stein et al. (2012) have been described above but another limitation is that it combined bulimic patients and EDNOS patients into one group stating, “No differences were found between the BN and EDNOS patients in any of the parameters assessed, enabling their inclusion in one group” (Stein et al., 2012, p. 617). Even though the study did not find any differences in measures between the two (except that the frequency of purging was a minimum of two weekly purging episodes for at least 6 months in the EDNOS group) and it is possible that if the diagnosis of the EDNOS group were done with the DSM-V it may have fallen under BN, it is still a heterogeneous group.

As the results of each study vastly vary a solid conclusion cannot be drawn. The overall findings of these five studies lean toward there being no differences in olfaction between BN patients and the general population. When looking at studies on traits that are present in bulimia, a study on impulsivity showed that highly impulsive females did not respond differently to olfactory stimulus (Larsen et al., 2012). Other studies on impulse related disorders also support similar outcomes. A study on olfaction in ADHD in adults demonstrated no significant differences (Schecklmann et al., 2011). However, it is necessary to reproduce more studies that investigate smell capacity in BN in a larger sample, improving on the limitations of previous studies.

### Obesity and overweight

Only two studies (Obrebowski et al., 2000; Fernández-Aranda et al., 2015) that examined smell capacity in obesity in humans were found and another study (Simchen et al., 2006) investigated smell in an overweight sample using the ETOC (Thomas-Danguin et al., 2003). In total, the Obesity studies had 89 patients and 116 healthy controls.

With the exception of 15 boys (16.9%) in Obrebowski et al. (2000), all participants were females. Low levels of olfactory threshold were detected in 7–41% of children.

The outcomes of Obrebowski et al. (2000) are difficult to generalize because it specifically looks at children between ages 10 and 16 (mean age of 12) and uses no control group. It is not clear whether the participants had an ED. Thus, it is possible that there were some participants with BN or BED or other eating pathologies in this group. In addition, an old instrument like the Elsberg–Levy's olfactometry (Pruszewicz, 1965), was used to measure olfaction. Considering the more updated olfactory tests available today, it would be interesting to know why the authors opted for using this relatively old fashioned instrument.

The Obesity group in Fernández-Aranda et al. (2015) excluded ED patients (such as patients who had BED). They were all outpatients seeking treatment for obesity. Fernández-Aranda et al. (2015) used the Sniffin' Sticks and analyzed all three variables of olfaction. The control group was divided into two: a younger sample [age range: 19–29, with mean 22.6 (2.9)], and an older sample [age range: 30–50, with mean 37.3 (5.9)]. However, this was done in order to avoid age as a confounding factor since the Obesity group was found to be significantly older [mean 37.5 (8.69)] than the AN group [mean 24.02 (5.32)]. In threshold, the Obesity group scored significantly lower than older controls and AN patients. Findings were similar for identification, where the obese group showed lower scores than younger controls, older controls and AN patients. 54.3% of the obese group had hyposmia (in the AN group the percentage was only 6.44%). The TDI from this last study showed that overall smell capacity in the obese group was significantly lower than in older controls and AN patients.

The total sample in Simchen et al. (2006) was 313. It was divided into two age groups: the ≥ 65 years group (that included participants who were 65 or older) and the < 65 years group (that included participants aged between 20 and 64). The scores for odor detection and odor identification were lower in participants with a BMI ≥ 28 kg/m2 than in participants with a BMI < 28 kg/m2 in the < 65 years group. However, in the ≥ 65 years group, scores were higher in participants with a BMI ≥ 28 kg/m2 than in participants with a BMI < 28 kg/m2 (13.8).

The findings are somewhat similar to that of the obesity studies considered in this review except that what is notable here is that threshold and identification are stronger in participants over age 65 than in younger ones when in cross-sectional non-ED studies on elderly population, olfactory capacity has been found to be impaired (Devanand et al., 2010; Mullol et al., 2012) and weight to increase with age (Gottlieb et al., 2014; Masurkar and Devanand, 2014). It is mentioned in Wilson and Morley (2003), that weight loss is observed after individual's 7th decade which could be due to loss of lean mass and fat mass but olfactory capacity is impaired.

Even though this study has a large sample, there was no control group involved. Respondents who were younger than 20 were rejected. Including younger participants and further dividing the < 65 years group into different age groups may have provided some relevant findings.

A human study even indicated toward olfactory dysfunction as a possible contributing factor to the progress of obesity (Richardson et al., 2004). The number of olfactory studies conducted on individuals with obesity is very limited. It can roughly be deduced from the three studies (Obrebowski et al., 2000; Simchen et al., 2006; Fernández-Aranda et al., 2015) found eligible for this review that since obese individuals have weak olfactory threshold and identification, their smell capacity is poor. Moreover, in Fernández-Aranda et al. (2015), which has a high sample power, the obese group showed an overall weaker olfactory capacity when compared to older controls. The prevalence of hyposmia in this group was 54.3%. This finding is further supported by Richardson et al. (2004) who did a study on obese and morbidly obese patients (even though it was not done in the required systematic way in order to be considered for the review) and found that participants with a BMI > 45 are more likely to show an olfactory dysfunction than participants with a BMI < 45.

Smoking habit ought to be taken into consideration as well because it could influence one's olfactory capacity (Hayes and Jinks, 2012). Another factor that is worth mentioning is the use of home-made olfactory tests in one of the studies (Stein et al., 2012). These tests have not been assessed for validity and thus their use as a valid instrument can be questioned. Even though it was controlled for in most studies presented in this review, pharmacological treatment may also influence olfaction as low olfactory threshold was observed in medicated participants (Lombion et al., 2008).

## Discussion

A total of 14 studies were included in this review. As the findings of each study greatly vary, a firm conclusion could not be drawn. Most ED studies on olfaction centered on AN. However, the overall findings seem to lean toward a weaker olfaction in AN even though Fernández-Aranda et al.'s (2015) study which presents the strongest results, observes a higher olfactory capacity in AN. The general findings of the five BN studies indicate toward there being little to no differences in olfaction between patients and the general population. Current evidence does imply that the sense of smell is weaker in the overweight and obese population.

This review focuses specifically on olfaction, especially its three aspects: threshold; discrimination and identification. If the review also included studies where the measures of olfaction were more general then this may have yielded some additional data. For example, if animal studies were included as a variable this could provide more information on the similarities and differences between humans and animals with regard to EDs and olfaction and their brain mechanisms. While on the one hand, it may be interesting to consider including studies that measured general olfactory capacity, on the other hand the exclusion of such studies keeps the focus on the more specific, in depth aspects of olfaction.

What is also apparent here is the lack of studies conducted on males. EDs are more prevalent in females but as findings have shown sex differences in olfaction (Doty and Cameron, 2009), more olfactory studies on males with ED could provide useful data in this field of research.

The findings of this systematic review show the methodological limitations of the research to date indicating a high level of heterogeneity in outcome. Due to the heterogeneity (differences in methodology, participants, measurements used and/or lack of literature in this review), it was not possible to conduct a meta-analysis. Differing results in the above-mentioned studies may be due to manifold (potentially confounding) factors such as gender, age, comorbidity, medication use, smoking habits (Hayes and Jinks, 2012), and testing instruments (Fedoroff et al., 1995; Kopala et al., 1995; Obrebowski et al., 2000; Lombion-Pouthier et al., 2006; Simchen et al., 2006; Schreder et al., 2008; Aschenbrenner et al., 2009; Rapps et al., 2010; Weiland et al., 2011; Goldzak-Kunik et al., 2012; Schecklmann et al., 2012; Stein et al., 2012; Dazzi et al., 2013). These factors ought to be controlled for in future studies. Studies would also largely benefit from the use of a control group.

Moreover, apart from Simchen et al. (2006) and Fernández-Aranda et al. (2015), all the studies have relatively low sample power. As such, the sample sizes (and heterogeneneity) may not be representative enough and this may be one of the reasons why the studies yielded different outcomes. They ought to be replicated with a larger sample which should be more clearly distributed. For example, considering the variable age, during the statistical analysis in the case of Simchen et al. (2006) the participants were only categorized into two age groups where one group included patients aged 20–64. This study overlooks the several possible differences between different age groups, ones that may have a confounding effect in the results, between the youngest participants and the older ones.

Olfactory disorders, such as anosmia or hyposmia, themselves are another factor that has not been controlled for in the control group of many of the studies. Functional anosmia and hyposmia have been found to occur in between 5 and 20% of the general population under age 65 (Landis et al., 2004; Mullol et al., 2012). This is particularly important in order to avoid any interpretational bias. It may further explain the different scores of the control group between each study such as in Fernández-Aranda et al. (2015) as stated earlier, where those variables were controlled for. When considering findings obtained in the hereby reported studies with clinical populations, in general terms, they mainly suggest that food-olfactory driven behaviors are clearly affected by the long-term metabolic status (starvation or malnutrition/excessive eating). In concordance with this assumption, studies with humans and animals have found that 24 h fasting can induce smell capacity (Cameron et al., 2012), whereas hyperlipidemic diet and hyperinsulinemia may cause impairment in the olfactory capacity (Lacroix et al., 2008; Thiebaud et al., 2014). This hints again toward ED and abnormal eating behaviors being a main influencing factor in olfactory dysfunction. However, it is not possible to distinguish whether olfactory impairment is state or trait finding and prospective design studies are here needed.

This is the first systematic review to be conducted on olfactory capacity in ED. The findings do indicate alterations of smell capacity in AN patients. However, the lack of research of olfaction in obesity, BN and BED is evident. Very little to no studies have been carried out with regard to olfaction in the overweight human population and people with other EDs such as BED. Yet, the findings of the above-mentioned studies, the crucial role the brain reward system plays in olfaction and eating (Frank et al., 2012) and animal studies showing olfactory dysfunction in abnormal eating (Tucker et al., 2012; Badonnel et al., 2014; Thiebaud et al., 2014) stress the need for more research. An animal study carried out by Tucker et al. (2012) suggests that the olfactory system may play a significant role in metabolism and feeding. Research on BN and AN require replication with a larger less heterogeneous sample taking the above-mentioned factors into account. With more homogeneous studies in AN, a meta-analysis can be conducted. Such studies should also be carried out with the Ob population using a control group. Future studies ought to take all the limitations mentioned in the previous section into account and use similar research and assessment procedures to make the studies replicable.

### Conflict of interest statement

The authors declare that the research was conducted in the absence of any commercial or financial relationships that could be construed as a potential conflict of interest.
